# Terahertz Pulse Generation from GaAs Metasurfaces

**DOI:** 10.1021/acsphotonics.1c01908

**Published:** 2022-03-29

**Authors:** Lucy L. Hale, Hyunseung Jung, Sylvain D. Gennaro, Jayson Briscoe, C. Thomas Harris, Ting Shan Luk, Sadhvikas J. Addamane, John L. Reno, Igal Brener, Oleg Mitrofanov

**Affiliations:** † Electronic and Electrical Engineering, University College London, London WC1E 7JE, U.K.; ‡ Center for Integrated Nanotechnologies, Sandia National Laboratories, Albuquerque, New Mexico 87123, United States; § Sandia National Laboratories, Albuquerque, New Mexico 87123, United States

**Keywords:** terahertz generation, surface nonlinearity, metasurface, shift current, optical rectification

## Abstract

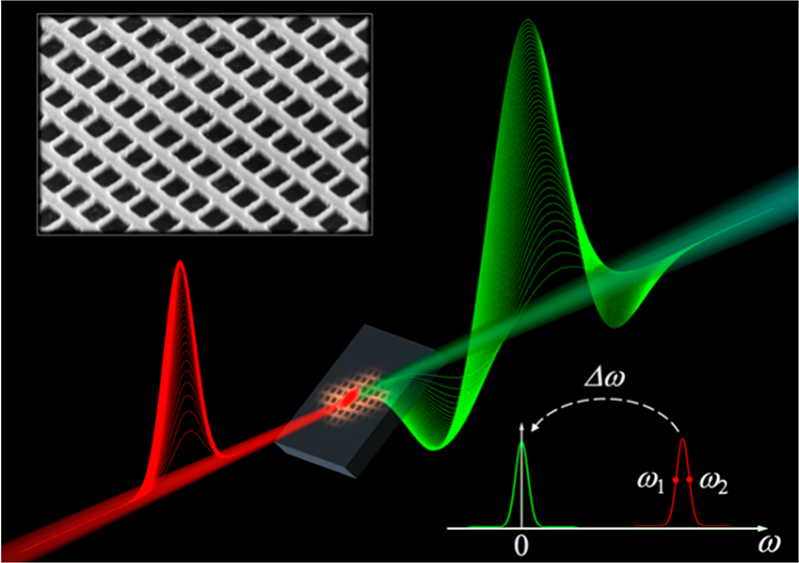

Ultrafast optical excitation of select materials gives rise to the generation of broadband terahertz (THz) pulses. This effect has enabled the field of THz time-domain spectroscopy and led to the discovery of many physical mechanisms behind THz generation. However, only a few materials possess the required properties to generate THz radiation efficiently. Optical metasurfaces can relax stringent material requirements by shifting the focus onto the engineering of local electromagnetic fields to boost THz generation. Here we demonstrate the generation of THz pulses in a 160 nm thick nanostructured GaAs metasurface. Despite the drastically reduced volume, the metasurface emits THz radiation with efficiency comparable to that of a thick GaAs crystal. We reveal that along with classical second-order volume nonlinearity, an additional mechanism contributes strongly to THz generation in the metasurface, which we attribute to surface nonlinearity. Our results lay the foundation for engineering of semiconductor metasurfaces for efficient and versatile THz radiation emitters.

Ultrafast optical excitation of materials using femtosecond pulses can generate a burst of terahertz (THz) radiation.^1−3^ This effect has enabled THz time-domain spectroscopy, a remarkably sensitive technique in the spectral range where efficient photonic sources are rare, and it unveiled the rich physics of the underlying process of THz pulse generation. Despite numerous studies, only a few materials, such as InAs,^1,4^ ZnTe,^5,6^ LiNbO_3_,^7^ and special material combinations such as W/Co_40_Fe_40_B_20_/Pt trilayers,^8−11^ have been found to possess the required physical properties that enable efficient generation of THz pulses. However, material limitations can be mitigated with optical metasurfaces. Metasurfaces composed of plasmonic resonators were recently developed to generate THz pulses through enhancement of a weak process of optical rectification at metallic surfaces.^12−17^ This process was further enhanced by integration of an epsilon-near-zero material underneath the resonators.^18,19^ However, optical rectification in common metals occurs only at the surface because of the inversion symmetry of the crystal lattice. As a result, metallic metasurfaces still suffer from low THz generation efficiencies in comparison with the established schemes involving nonlinear dielectrics and semiconductors, such as optical rectification in phase-matched ZnTe crystals^5,6^ and transient photocurrents in low-band-gap InAs.^1,4,20,21^

Semiconductor optical metasurfaces therefore could potentially outperform their metallic counterparts. Still, no all-dielectric metasurfaces for THz generation have been reported to date. Major challenges for semiconductor metasurfaces come from the metasurface geometry, which can diminish some of the THz generation mechanisms. THz generation by transient photocurrents requires that the charge carriers move in a preferential direction. In optically thick materials, built-in surface fields and photocarrier density gradients define this direction and enable a net photocurrent. In an ultrathin metasurface, however, these effects can be reduced and even cancel out completely. THz generation by optical rectification is also limited in a dielectric metasurface: while high-quality factor resonances enhance the field strength, they also limit the THz pulse bandwidth. These challenges therefore raise a question: can a semiconductor metasurface be used for THz pulse generation with efficiency comparable to that of bulk crystals?

Fortunately, semiconductor metasurfaces can take advantage of a broad range of THz generation mechanisms. Among them is optical nonlinearity, which is primarily caused by shift currents for above-the-band-gap excitation.^22−26^ The shift currents arise from a shift of charge distribution within the crystal lattice as a result of electron excitation from the valence band to the conduction band.^24,26^ This process should occur both in the material volume and at the surface, where the material discontinuity and surface states can alter the nonlinear characteristics.^27−30^ Although surface nonlinearity has not been identified as a THz generation mechanism to date, it has previously been shown to enhance second harmonic generation (SHG) in all-dielectric metasurfaces.^31−34^ The strong enhancement of these processes in all-dielectric metasurfaces therefore suggests that surface nonlinearities could also play a role in THz generation because of the high surface area to volume ratio.

In this work, we demonstrate the generation of THz pulses from a 160 nm thick GaAs metasurface excited above the material band-gap with an efficiency comparable to optically thick GaAs crystals. Our study points toward surface nonlinearity as a significant component in the THz generation process. The generated THz field exhibits a distinctive amplitude and polarity dependence on the polarization of the optical excitation, indicating that the dominant underlying mechanism is shift currents. However, through detailed analysis we find that shift currents in the metasurface volume do not fully account for the observed THz emission and that a significant portion of the THz field is likely to be generated at the surfaces. To our knowledge, this is the first observation of THz generation from an all-dielectric optical metasurface and the first evidence for a significant contribution of surface optical nonlinearity to the THz generation process. These results lay the foundation for the development of a versatile semiconductor metasurface platform for THz generation and show the potential for generating stronger THz pulses with higher efficiency.

## Experimental Results and Discussion

To determine whether surface nonlinearity plays a role in THz pulse generation, we first examined THz generation from a simple optically thin layer of GaAs, a semiconductor that supports several known THz generation mechanisms.^24,28^ For above-the-band-gap excitation by 100 fs optical pulses, we found that THz generation from the GaAs layers does not scale with the layer thickness and that the generated THz field strongly depends on the excitation polarization. The former finding indicates the presence of surface effects, whereas the latter points to optical nonlinearity as the underlying mechanism.

**Figure 1:**
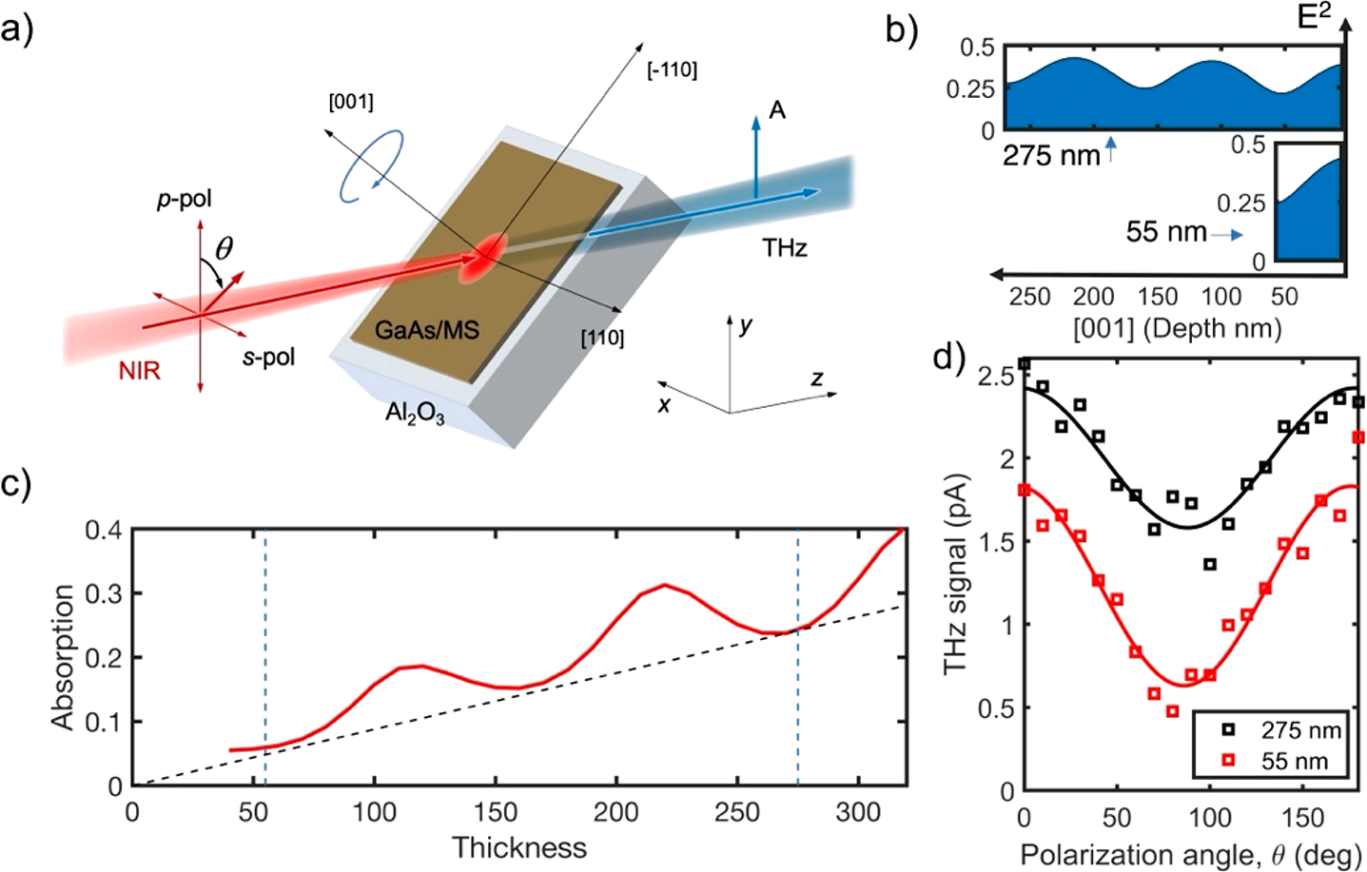
THz pulse generation in thin GaAs layers. (a) Schematic of the experimental system: pulsed near-infrared (NIR) light with polarization angle θ is incident on the sample (red arrows). A THz beam is emitted in the forward direction (blue arrow) and detected by a THz antenna with vertical orientation (*y* direction), marked as “A”. (b) Numerically calculated electric field intensities (*E*^2^) inside GaAs layers with thicknesses of 55 and 275 nm for photoexcitation at λ = 780 nm (normalized to the incident intensity). (c) Numerically calculated total absorption in a GaAs layer as a function of layer thickness. The approximate linear scaling of the absorption with thickness is shown for layers with similar standing wave distributions. (d) Peak-to-peak amplitude of generated THz pulses (p-polarized) for the two GaAs layers (55 and 275 nm thick) as functions of the excitation polarization angle θ (θ = 0° and 180° correspond to p-polarized excitation, and θ = 90° corresponds to s-polarized excitation).

The THz generation setup and results of these experiments are illustrated in Figure 1, where we compare THz pulse generation from two GaAs layers with thicknesses corresponding to λ/4 (55 nm) and 5λ/4 (275 nm) of the excitation wavelength (λ = 780 nm). These thicknesses are specially chosen because the two cases have similar electric field intensities at the GaAs–air interface (Figure 1b), whereas the numbers of generated photocarriers within the two layers are significantly different because of the difference in their respective thicknesses (Figure 1c). As a result, the surface-related contributions to the THz field should remain the same, whereas the volume-related contributions should scale with the layer thickness. Figure 1b shows the numerically calculated optical field intensities (*E*^2^) inside the GaAs layers for illumination at an angle of incidence of 45°, which is close to the optimum angle for THz generation.^2^ The intensity profiles display standing wave patterns inside the layers with peaks at the back interface and minima at the front interface in both cases.

Figure 1c shows that the total light absorption within the 55 nm layer is smaller by a factor of ∼4 in comparison with the 275 nm layer. However, the amplitude of THz pulses generated from these two GaAs layers upon photoexcitation varies by only a factor of 1.3. The THz pulse amplitude also exhibits a strong dependence on the excitation polarization angle, with the strongest THz pulses generated for p-polarized excitation (Figure 1d; full time-domain waveforms and experimental details are provided in the Supporting Information).

These observations allow us to identify the THz generation mechanisms at play in optically thin GaAs layers. THz generation from optically thick GaAs is primarily attributed to photocurrents driven by built-in surface fields.^35,36^ In addition, GaAs supports photocurrents caused by photoexcited charge carrier density gradients, leading to what is known as the photo-Dember effect.^35,37,38^ Finally, GaAs exhibits a relatively high second-order nonlinearity. Among these generation mechanisms, only the optical nonlinearity can show a strong dependence on the polarization of the optical excitation, as we observe for the 55 nm layer. The contribution of the nonlinear polarization to the THz signal in fact is expected to switch sign as the incident beam polarization is changed from p-polarized to s-polarized.^5^ While some polarization dependence is a result of Fresnel reflection at the interface, it accounts for only ∼10% of the change in THz amplitude. Therefore, neither the photocurrent due to built-in fields nor the photo-Dember current can explain the strong polarization dependence in Figure 1d. The experimental results strongly suggest that second-order nonlinearity is the dominant mechanism for the 55 nm GaAs layer. However, this process would occur throughout the volume of an optically thin layer, and thus, the THz pulse amplitude would scale with the layer’s thickness. The lack of such scaling for the 55 and 275 nm thick samples indicates that a significant portion of nonlinear THz emission must be generated at the GaAs surface. As we mentioned earlier, the leading nonlinear mechanism in GaAs for the above-the-band-gap excitation is shift currents.^23^ Therefore, shift currents—particularly at the surface—play an important role in THz generation from thin GaAs layers and thus from GaAs metasurfaces.

We next aimed to enhance THz generation by nanostructuring GaAs as a metasurface with enhanced optical absorption to induce shift currents. We designed a metasurface to exhibit degenerate critical coupling, which enables full (100%) absorption of optical excitation at a desired wavelength.^39^ In addition, to maximize shift currents, we designed our metasurface for an incidence angle of 45°, where the crystallographic axes of (001) GaAs do not coincide with the polarization of the incident optical field.^1^ With these considerations in mind, we developed a 160 nm thick GaAs metasurface design with complete absorption of s-polarized excitation at 780 nm and enhanced (∼60%) absorption of the p-polarized excitation.

**Figure 2:**
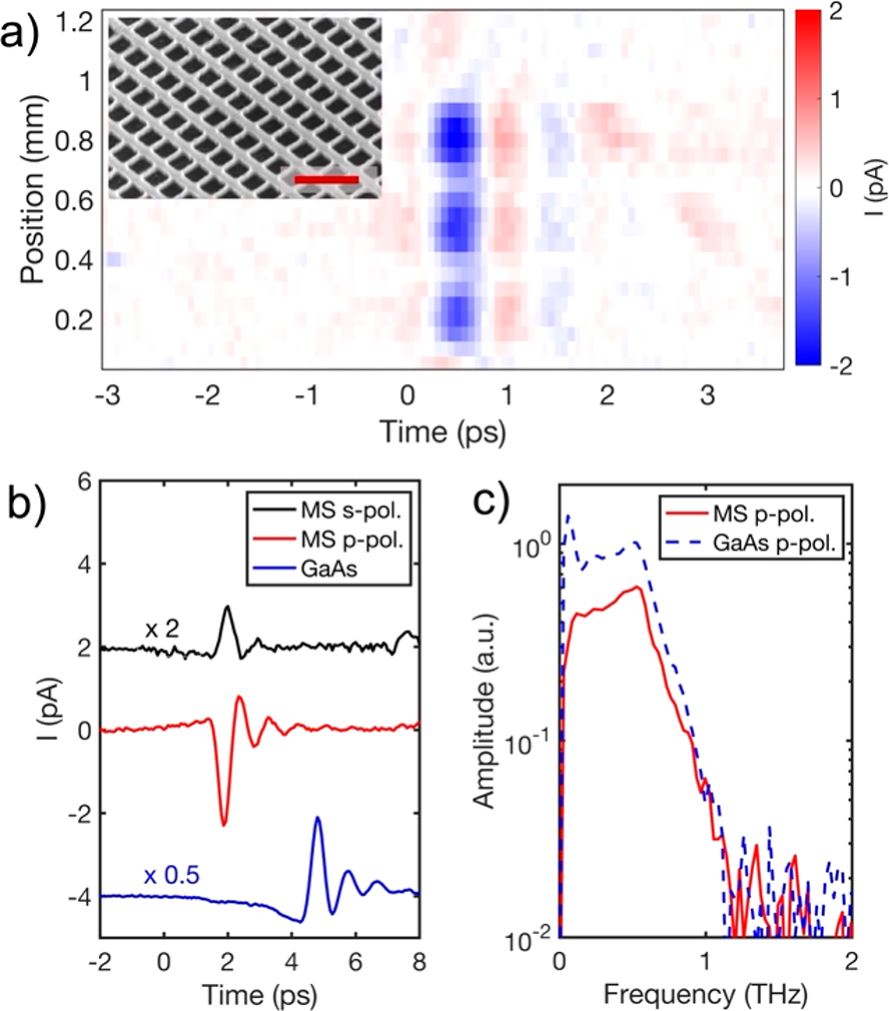
(a) THz field generated upon excitation at different positions in a sample containing three metasurfaces (MSs) with different wide bar widths: 100, 110, and 120 nm. THz generation is enhanced (bright blue spots) when the metasurfaces are excited at the center. The inset shows an SEM image of the metasurface at oblique angle (the scale bar is 1 μm). (b) Time-domain waveforms of THz pulses generated in an MS excited with s polarization (black, scaled by a factor of 2) and p polarization (red) compared with a THz pulse generated in a GaAs crystal (thickness = 650 μm) (blue, scaled by a factor is 0.5). (c) Fourier amplitudes of THz pulses for the MS and the thick GaAs crystal for p-polarized excitation.

Complete light absorption is achieved by critical coupling of the incident wave to two degenerate in-plane electric and magnetic dipole modes.^40,41^ We previously developed a GaAs metasurface based on this concept for normal-incidence illumination.^42,43^ Here, however, we modified that design to maximize absorption at an incident angle of 45°. A scanning electron microscopy (SEM) image of the developed metasurface is shown in Figure 2. The metasurface was transferred onto a sapphire substrate for experimental testing. More information on the metasurface design, fabrication, and optical properties can be found in the Supporting Information.

We illuminated the metasurface using 100 fs optical pulses and observed THz pulse generation using the configuration in Figure 1a. The electric field of THz pulses emitted in the forward direction was detected using a THz time-domain spectroscopy (THz-TDS) setup with a photoconductive antenna (PCA) oriented vertically and positioned ∼8 mm away from the metasurface.

The THz generation experiments yielded two noteworthy observations: first, the photoexcited metasurface emits THz pulses with a significantly higher amplitude compared with an unpatterned GaAs layer of the same thickness. Second, the polarity of the THz pulse is opposite to that emitted from the unpatterned GaAs layer. To illustrate these observations, in Figure 2 we show a line scan taken across a sample containing three similar metasurfaces on an otherwise unpatterned 160 nm thick GaAs layer. At each position along the scan, a time-domain waveform of the emitted THz field was recorded. The spatial and temporal data are presented in full as a space–time map (Figure 2a). The blue spots on the map correspond to the first oscillation of the THz field emitted from each of the three metasurfaces. In the areas where the metasurfaces are present the THz emission is strongly enhanced: the THz pulse amplitude is over 4 times higher than that emitted by the unpatterned GaAs layer, and the polarity of THz field is reversed. As well as allowing us to compare the metasurface with the unpatterned GaAs layer, Figure 2a shows that the polarities and temporal profiles of the THz pulses emitted from the three metasurfaces are the same. The THz pulse amplitude, however, depends on the metasurface dimensions (see the Supporting Information for more detail).

Remarkably, the THz emission from the 160 nm thick metasurface is comparable in amplitude to the emission from a much thicker GaAs crystal (650 μm), despite the significantly reduced available material volume in the metasurface. Interestingly, the emission from the thick crystal has the opposite polarity relative to the metasurface (Figure 2b). Apart from the opposite polarity (which corresponds to a phase shift of π), the spectra of the detected pulses are similar (Figure 2c).

The change in pulse polarity indicates that different generation mechanisms are at play. The photocurrent generation mechanism—commonly accepted as the leading mechanism for THz generation in GaAs—must play a smaller role in the metasurface. Evidence for this can be seen from the dependence of the THz pulse polarity on the polarization of the laser excitation for the metasurface: the polarity is reversed when switching between p- and s-polarized excitation (black trace in Figure 2b). This is not possible if photocurrent is the main generation mechanism, as the photocurrent direction is not dependent on the excitation polarization. Furthermore, THz pulses with 3 times higher amplitude are generated when the metasurface is excited with p-polarized light even though only ∼60% of the photocarriers are excited in comparison with the case of s-polarized light (see the Supporting Information). If photocurrent were the main generation mechanism, the pulse amplitude would be smaller for the p-polarized excitation rather than significantly higher since the metasurface absorbs less at this polarization. The above observations strongly suggest that second-order optical nonlinearities dominate THz generation in this GaAs metasurface.

Next, we address the question of the origin of this nonlinearity: is it within the metasurface volume or at the surface? Nonlinear polarization (shift currents) in the metasurface volume can be evaluated numerically. However, very little is known about THz generation due to shift currents at the surface, even for plane GaAs. A metasurface therefore presents a very challenging problem: first of all, it contains surfaces with several orientations, and second, the optical fields contain all three vectoral components in the metasurface. In addition, it is likely that the nonlinear polarization is highly sensitive to the quality of the interfaces and surface roughness, although little is known about this effect. A quantitative evaluation of surface nonlinearity in the metasurface by means of numerical modeling would require a verified model, which has not been developed yet. Nevertheless, we can evaluate the polarization dependence of the induced THz field due to the second-order nonlinearity within the metasurface volume and compare it to experimental observations.

**Figure 3:**
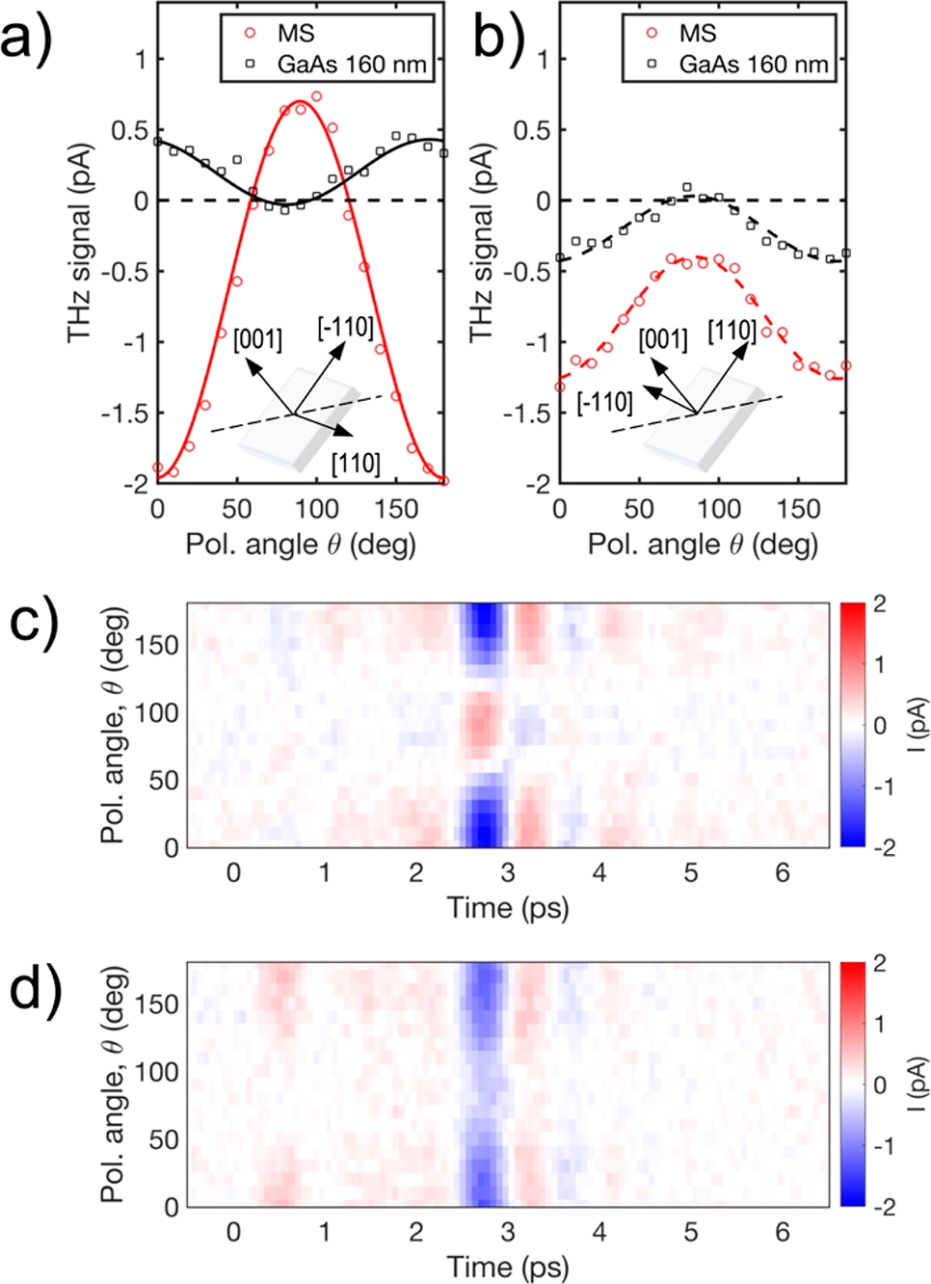
Polarization dependence of the radiated THz field. (a, b) Peak-to-peak THz fields measured for varying excitation polarization angles for the metasurface (MS, red) and a GaAs layer of the same thickness (160 nm) (black) for two orientations of the sample (see the insets). (c, d) THz field maps showing the THz generation from the metasurface for varying polarization angle θ, for two orientations of the sample: (c) orientation shown in the inset of (a); (d) orientation shown in the inset of (b).

In Figure 3 we provide measurements of the amplitude of the THz pulses emitted from the metasurface for a range of polarization angles θ and for two orientations of the sample. The THz pulse amplitude is maximum (negative value) when the excitation is p-polarized (θ = 0° and θ = 180°). As we rotate the excitation polarization, first the THz pulse amplitude decreases, and then the pulse polarity is reversed and the amplitude exhibits a local maximum for s-polarized excitation (Figure 3a). Figure 3b shows how the THz generation changes when the sample is rotated by 90° about the surface normal. In this case, no change in polarity is seen when the polarization is rotated, yet the THz pulse amplitude still strongly depends on θ, with a maximum observed for p-polarized excitation and a minimum for s-polarized excitation. Full time-domain THz pulse waveforms are shown in Figure 3c, where one can see that the shape of the emitted THz pulse remains unchanged and only the THz field amplitude and polarity vary with the excitation polarization.

**Figure 4:**
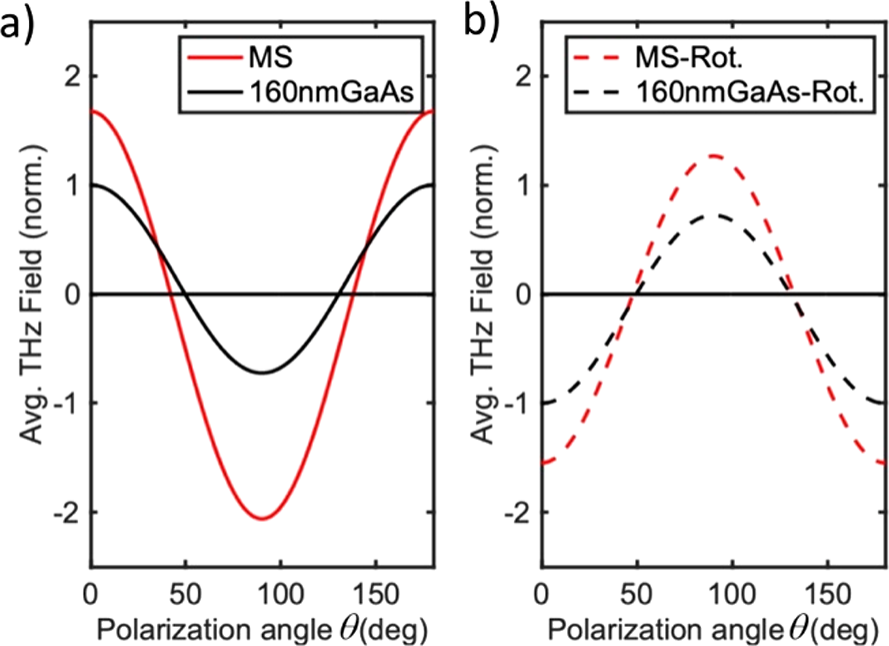
(a) Calculated amplitudes of the THz fields generated by second-order nonlinearity in the metasurface volume (red) and in the volume of a GaAs layer of the same thickness (160 nm, black) as functions of the excitation polarization angle θ. (b) Calculated field amplitudes for the samples rotated by 90°. The field amplitudes for both the metasurface and thin layer are normalized to the maximum field for the thin layer (θ = 0).

We now compare the experimentally measured THz field dependence on the polarization angle to the numerically calculated bulk second-order nonlinear polarization. In Figure 4 we provide the calculated average nonlinear polarization *P*_*y*_ induced in the metasurface using the numerically simulated electric field distribution (for detailed calculations, see the Supporting Information). The average nonlinear polarization and the corresponding THz field amplitude are expected to switch polarity for s- and p-polarized excitations, and in both cases the THz pulses are expected to have similar absolute amplitudes. Our experimental results are in stark contrast to these predictions: the THz pulse amplitude for p-polarized excitation is ∼3 times larger than for s-polarized excitation in Figure 3a. Furthermore, when the crystal is rotated by 90°, the THz field polarity is expected to flip, while the absolute amplitudes remain similar for s- and p-polarized excitations. Again, the experimental data show a different behavior: the polarity remains the same, and the THz field amplitude is significantly reduced for s polarization.

The observed dependence of the THz pulse amplitude on angle θ cannot be explained by the second-order nonlinearity arising from within the metasurface volume. Nor can it be explained by adding effects of drift photocurrents (as previously discussed). In addition, the photocurrent direction should be independent of the crystal orientation, meaning that it cannot account for the dependence of the polarity on the crystal orientation either. To emphasize this point, we also show in the Supporting Information that even in the simple case of an unpatterned thin GaAs layer it is impossible to account for the observed THz field dependence using the volume nonlinearity and photocurrent mechanisms alone.

We therefore conclude that THz pulse generation from the metasurface originates not only from the volume but contains contributions from another mechanism. Our earlier analysis of thin GaAs layers (Figure 1) suggests that the surface nonlinearity is a likely candidate for this mechanism. Not only is the surface nonlinearity expected to strongly depend on the excitation polarization, but it may also be influenced by the surface orientation.

**Figure 5:**
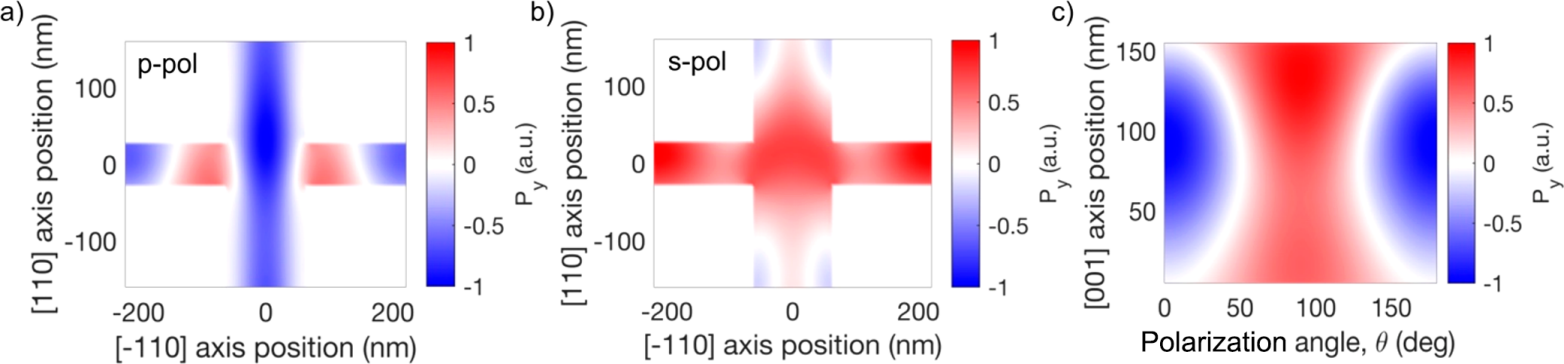
(a, b) Spatial distributions of the induced nonlinear polarization *P*_*y*_ in the metasurface plane (averaged over the metasurface depth) for (a) p-polarized and (b) s-polarized excitation. (c) Nonlinear polarization in the metasurface (averaged over the metasurface plane) as a function of the incident light polarization angle θ and the metasurface depth (distance along the (001) axis).

It is important to note that the induced nonlinear polarization and as a result the THz field are highly dependent on the metasurface design. To illustrate this, we show in Figure 5 the spatial distribution of the induced nonlinear polarization component *P*_*y*_ within the metasurface. In the *xy* plane (Figure 5a,b), the distribution of *P*_*y*_ in our metasurface unit cell is predominantly negative for p-polarized optical excitation and positive for s-polarized optical excitation. Such distributions, showing the same sign within the unit cell, add up coherently to emit THz radiation. In each case, however, there are small regions with *P*_*y*_ of the opposite sign, which diminish the total radiated THz field.

Furthermore, Figure 5c shows that the nonlinear polarization is maximum near the metasurface central plane (*z* ≈ 80 nm) for the p-polarized excitation, whereas the nonlinear polarization is stronger at the back surface for the s-polarized excitation. Although there is no verified model from which we can calculate the surface nonlinear polarization contribution in the same way as shown for the volume contribution, we can expect variation of surface nonlinear polarization across the unit cell, with regions of both positive and negative contributions. As a result, it is important to design a metasurface not only with maximum absorption but also with a spatial field distribution that contributes constructively to the THz generation process. Further studies of other metasurface designs can provide needed insight into the role of the electric field distribution in the contributions of volume and surface nonlinearity to THz generation.

## Conclusion

We have demonstrated that ultrathin GaAs metasurfaces can generate THz radiation with absolute field amplitudes comparable to those of thick bulk crystals but with polarities that depend on the excitation beam polarization. To our knowledge, this is the first demonstration of THz generation from an all-dielectric metasurface. While photocurrent and shift currents are cited as the main THz generation mechanisms in optically thick GaAs, we have shown that explaining the observed enhanced THz generation in our GaAs metasurface requires a third, alternative mechanism, which we identify as shift currents at the surface. Ultrathin semiconductor metasurface-based THz emitters could be designed to potentially exceed the THz generation efficiencies of bulk semiconductors and allow control of the emitted THz field amplitude and phase. However, more investigation is needed to untangle the contributions of the different THz generation mechanisms and to understand how to exploit surface nonlinearity to boost the THz generation process. This work lays the foundation for high-efficiency, tailored, and reconfigurable metasurfaces for THz generation.

## Supporting Information Available

Numerical simulations, fabrication methods, and experimentally measured optical properties of the metasurface; experimental THz time-domain spectroscopy setup and full time-domain waveforms of GaAs thin layers; detailed calculations of second-order nonlinear polarization in the metasurface and thin-layer volume; analysis of THz generation mechanisms in a thin GaAs layer (PDF)
