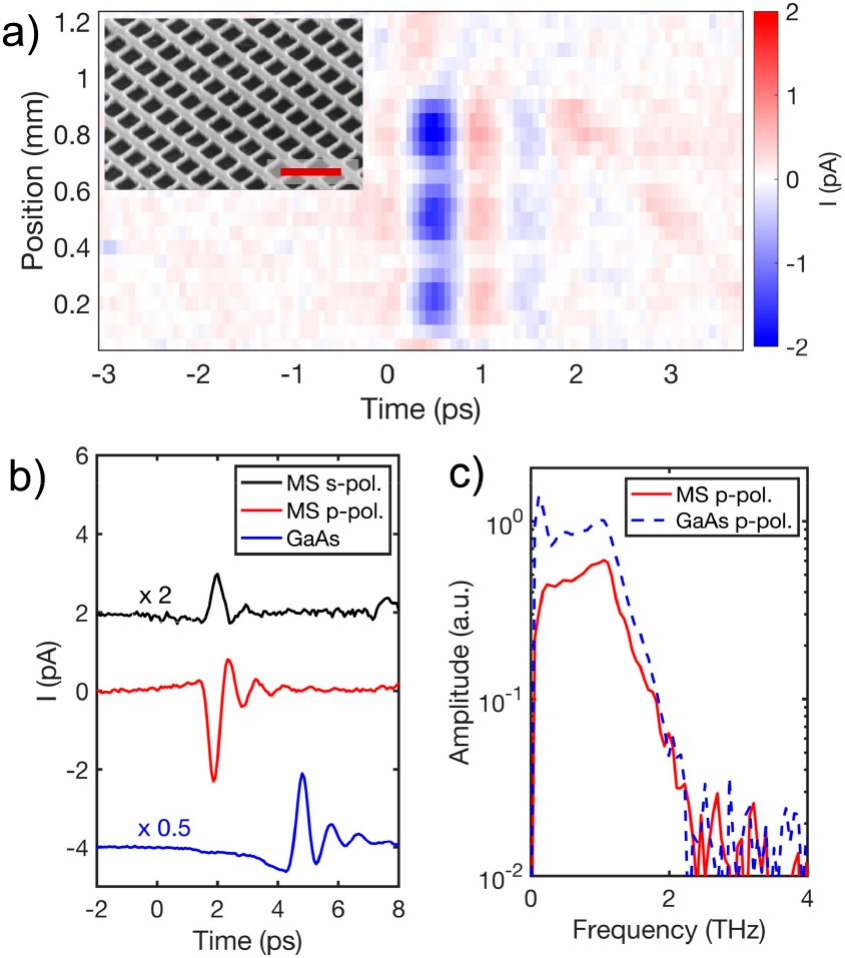# Correction to “Terahertz Pulse Generation from GaAs Metasurfaces”

**DOI:** 10.1021/acsphotonics.2c00878

**Published:** 2022-06-29

**Authors:** Lucy L. Hale, Hyunseung Jung, Sylvain D. Gennaro, Jayson Briscoe, C. Thomas Harris, Ting Shan Luk, Sadhvikas J. Addamane, John L. Reno, Igal Brener, Oleg Mitrofanov

In the previous manuscript, the horizontal axis in Figure 2c was incorrect (showing [1,2] THz when it should have showed [2,4] THz). It is important to correct this error in order to correctly present the experimental results and the bandwidth of the metasurface. This was simply a typographical error and nothing else about the manuscript text has been changed.

Corrected figure: