# Supplementary material for: Terahertz Pulse Generation from GaAs Metasurfaces
Source: ACS Photonics. 2022 Mar 29;9(4):1136–42. doi: 10.1021/acsphotonics.1c01908 (PMC9097576; doi:10.1021/acsphotonics.1c01908)
Supplement: Supplementary file 1 — ph1c01908_si_001.pdf [file ph1c01908_si_001.pdf]

Terahertz pulse generation from GaAs metasurfaces

Lucy L Hale[1], Hyunseung Jung[2,3], Sylvain D Gennaro[2,3], Jayson Briscoe[2,3], C. Thomas Harris[2,3], Ting Shan Luk[2,3], Sadhvikas J Addamane[2,3], John L Reno[2,3], Igal Brener[2,3], Oleg Mitrofanov[1,2]

[1]*University College London, Electronic and Electrical Engineering, London WC1E 7JE, UK*
[2]*Center for Integrated Nanotechnologies, Sandia National Laboratories, Albuquerque, New Mexico 87123, USA*
[3]*Sandia National Laboratories, Albuquerque, New Mexico 87123, USA*

Supporting Information

1. Metasurface Design

1.1 Numerical simulations

The GaAs metasurface is designed and simulated using a commercial finite difference time-domain (FDTD) software (Lumerical) [1]. A single unit cell of the metasurface is modelled with perfectly matched layer (PML) boundaries above and below the metasurface. The GaAs metasurface is submerged in a dielectric substrate with the refractive index of n = 1.57 and no material losses. The top surface of the substrate and the top surface of the metasurface coincide replicating the geometry of the fabricated metasurfaces bonded to sapphire substrates by epoxy. Metasurface fabrication is described in Section S2.

A plane wave source is launched from within the substrate and directed at an angle β^* to the metasurface normal. This angle relates to the angle of incidence β in Figure S1 as $n \sin\beta = \sin\beta^*$. It replicates the incidence angle after refraction through the air/sapphire and sapphire/epoxy interfaces and before light reaches the metasurface. The broadband fixed angle source technique (BFAST) [2,3] and the corresponding boundary conditions are used in order to maintain a constant illumination angle

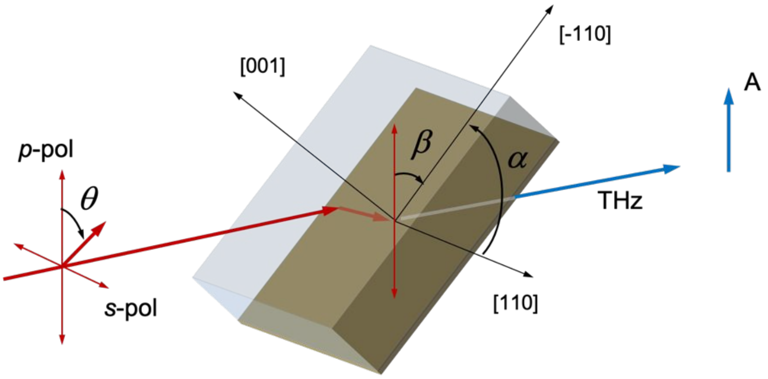

Figure S1. Schematic diagram of metasurface excitation geometry: polarization of the incident field is given by θ, the angle of incidence β = 45°, the angle of sample rotation is α is (α = 0° or 90° in the experiment). The THz field is measured in the vertical polarization labeled by A (p-polarized).

across the simulation bandwidth. Absorption (A) in the metasurface is calculated from the simulated reflection (R) and transmission (T) coefficients, as A = 1- (R+T).

1.2 Optical absorption properties of metasurface

The metasurface is designed to achieve full absorption for *s*-polarized illumination at 45° to the metasurface plane using the concept of degenerate critical coupling [4]. The design is adapted from a metasurface reported in [5], which achieves perfect absorption for normal incidence excitation. The dimensions of this metasurface are shown in Figure S2a, and Figure S2b shows the simulated optical properties with perfect absorption at 775 nm.

The two modes used for degenerate critical coupling are the electric dipole oriented in the *x*-direction and magnetic dipole oriented in the *y*-direction. The simulated electric and magnetic field distributions for these modes are shown Fig. S2c,d respectively.

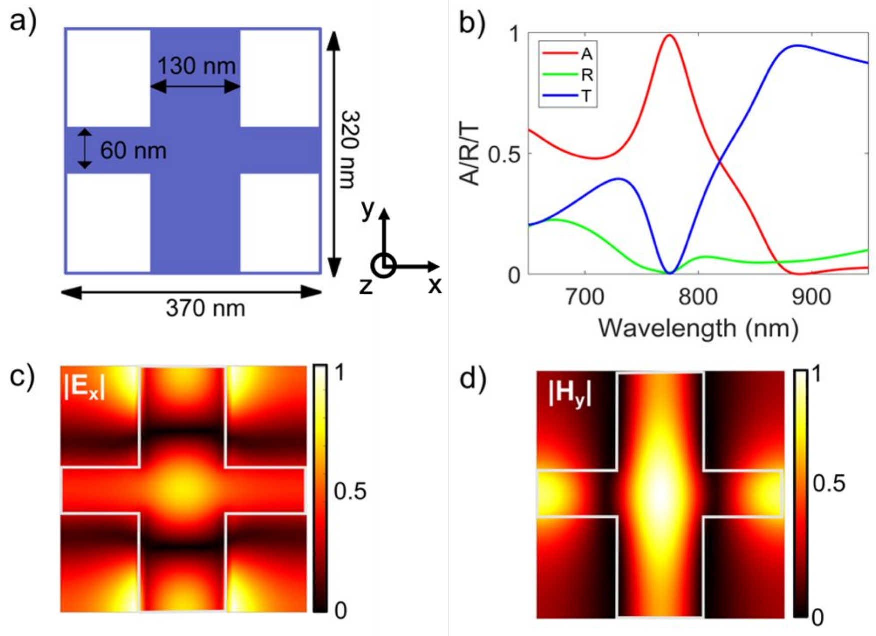

Figure S2. Design of the perfectly absorbing GaAs metasurface: **a)** Schematic diagram of the metasurface unit cell proposed in ref. [5]. **b)** Simulated optical properties of metasurface: Absorption (A), Reflectance (R), Transmission (T). Cross section of the metasurface unit cell showing **c)** absolute electric field distribution (polarized in the *x*-direction, see panel a); and **d)** absolute magnetic field distribution (polarized in the *y*-direction, see part a) at 775 nm.

In Figure S3a, the simulated absorption spectra of the metasurface are shown for varying angle of incidence. The angle is changed from normal incidence (β = 0°) to β = 51° by tilting the metasurface

plane with respect to the optical axis such that the *y*-axis of the metasurface remains in the plane of incidence (Fig. S1).

When the angle of incidence is increased, the two modes shift at different rates to shorter wavelengths and mode degeneracy is lost. However, at an angle of incidence of 45° (corresponding to the incidence angle used in the experiment), perfect absorption can be regained by increasing the metasurface periodicity in the *x*-direction. This is shown in Fig. S3b, where the metasurface periodicity in the x-direction is increased from 320 nm to 440 nm. At a periodicity of 440 nm, mode degeneracy is reached and, as a result, the metasurface exhibits near perfect absorption at 800 nm for *s*-polarized light (marked by black circle in Figure S3b). Figure S4 shows the dimensions and simulated optical properties of the new metasurface design optimized for the incidence angle of 45°.

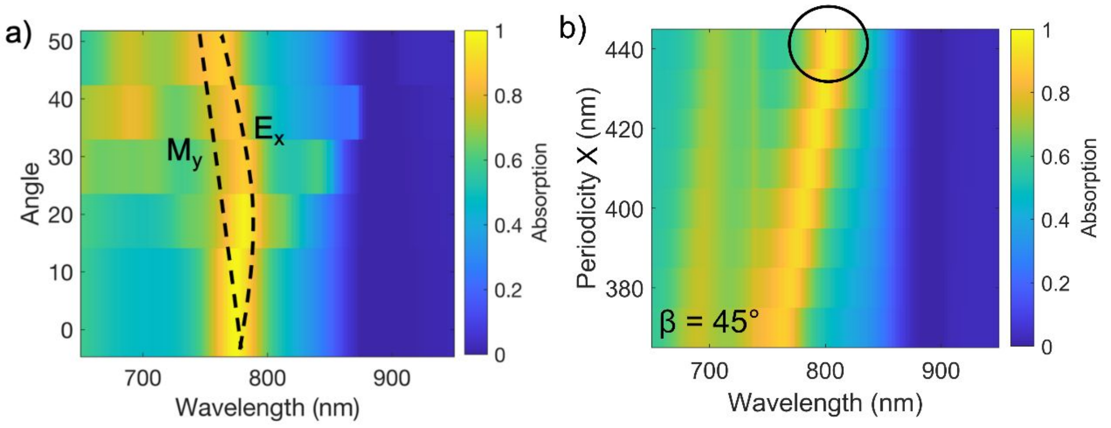

Figure S3. Absorption spectra of the metasurface for oblique incidence: **a)** Absorption map illustrating spectral position of the modes for the incidence angle ranging from 0° (normal incidence) to 51°. **b)** Absorption map illustrating the wavelength shift of enhanced absorption band for different x-periodicity when the angle of incidence is 45° (the corresponding angle of incidence from inside the substrate (epoxy) β^* = 28°).

Different modes are excited when the metasurface is illuminated by p-polarized light with the wavelength of 800 nm: the electric dipole mode oriented in the *y* direction (in-plane) and the electric dipole oriented in the *z* direction (out of plane). While these two modes overlap, they are not degenerate and perfect absorption is not achieved. Nevertheless, absorption is enhanced to ~ 55% (see Fig. S4b), compared to ~17% in a uniform GaAs layer of the same thickness.

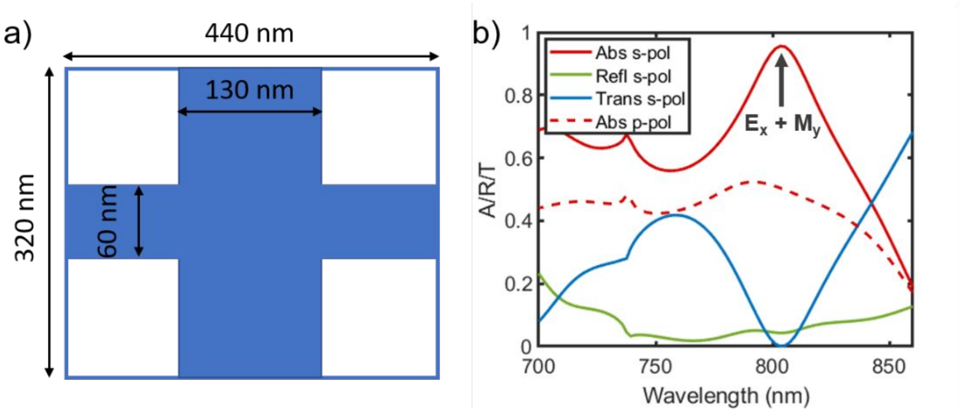

Figure S4. Design and optical properties of perfectly absorbing metasurface for the angle of incidence of 45°: **a)** Metasurface unit cell dimensions. **b)** Absorption (red), reflectance (green) and transmission (blue) for s-polarized illumination and absorption (red dashed) for p-polarized illumination. The position of the degenerate electric dipole E_x and magnetic dipole M_y modes are shown by the black arrow.

2. Metasurface Fabrication

Metasurfaces are fabricated using a 160 nm thick GaAs layer grown by molecular beam epitaxy at 250º C. Prior to the metasurface layer growth, two $Al_{0.55}Ga_{0.45}As$ stop-etch layers with a 100 nm thick GaAs spacer are grown on a semi-insulating (100)-oriented GaAs wafer (wafer number VA1013). The samples are annealed at 600° C for 40 sec in forming gas.

300 μm X 200 μm metasurface patches are defined on the epilayer surface using electron-beam lithography (EBL) and the positive tone photoresist (ZEP). The metasurface lattice vectors *x* and *y* are oriented along the [110] and [-110] directions of the GaAs lattice, respectively. Samples with developed photoresist patterns are then etched in an inductively coupled plasma (ICP) reactive ion etching (RIE) system to the depth of ~160 nm using a mixture of BCl_3, Cl_2, Ar and N_2 gases (10, 10, 10 and 3.5 cm^3/min respectively). The radio frequency (RF) power applied to the sample chuck during the etch is 500 W. The photoresist is then removed in Remover PG (at 80° C). A scanning electron micrograph of a typical etched metasurfaces is shown in Figure S5.

The samples are then bonded to a 0.5 mm thick c-cut sapphire substrate using a ~2 μm thick layer of epoxy (Epotek 353ND). The SI-GaAs substrate is removed using mechanical lapping first, followed by wet etching. A citric acid etch (citric acid : H_2O_2 – 5 : 1) is used to etch the remaining GaAs and a phosphoric acid etch (H_3PO_4 : H_2O : H_2O_2 – 20 : 200 : 4) is used to remove the first $Al_{0.55}Ga_{0.45}As$ stop-etch layer. The wet-etch process is then repeated to remove the GaAs spacer layer and the second

stop-etch layer. At the end, only the 160 nm thick GaAs layer with several metasurface pattern remains bonded by the epoxy to the sapphire substrate.

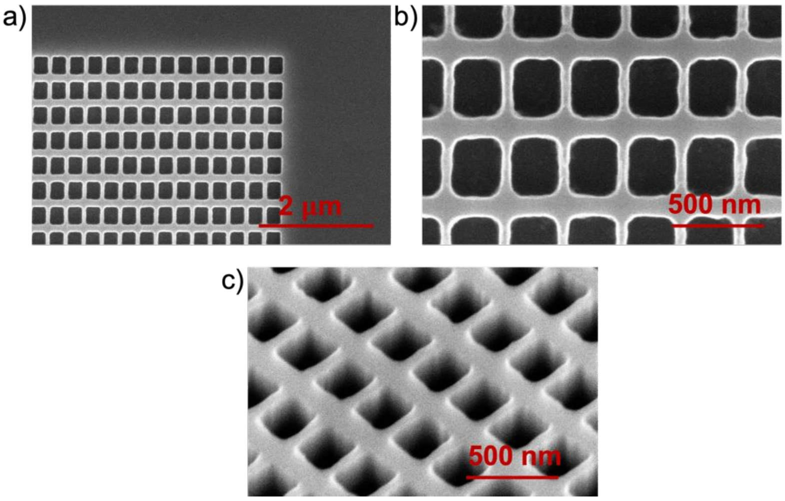

Figure S5. Scanning Electron Microscope (SEM) images of the (a) metasurface corner and (b,c) central region: (b) top view, (c) oblique angle showing metasurface side walls.

3. Metasurface Optical Properties

In order to optimize degeneracy of the two modes experimentally, metasurfaces with different bar widths are fabricated and their optical transmission properties are measured for different angles of incidence using a confocal microscope set-up. White light from a fiber-coupled incandescent lamp is passed through a Glan-Thompson polarizer and focused on the metasurface with an NA = 0.4 microscope objective to a spot size of ~100 μm. The transmitted beam is collected by another microscope objective (NA=0.3) and analyzed with a diffraction grating spectrometer. The angle of incidence is varied from 0 ° to 45° by tilting the metasurface with respect to the optical axis. Metasurface spectra for each angle of incidence are normalized to that of the sapphire substrate without the metasurface.

Figure S6a illustrates typical transmission spectra for the incidence angle of 45°. The effect of enhanced absorption results in a strong drop in transmission [6], In each spectrum, two dips in transmission are clearly visible. The first dip at 760nm (780nm) corresponds to the two overlapping modes, E_x and M_y. Transmission decreases to ~10% at mode wavelength and absorption thereby increases. The dip at 870 nm (885nm) corresponds to the out-of-plane magnetic dipole mode M_z, which is only excited for non-normal incidence excitation, as seen in the transmission map in Fig. S6b. The map also shows that low transmission (and therefore high absorption) due to the two overlapping modes

is achieved over a range of angles from ~30° to 45°. For smaller angles of incidence, the low transmission band becomes broader with higher values of transmission. This is caused by the overlapping modes moving spectrally apart (Fig. S6a) and reducing absorption.

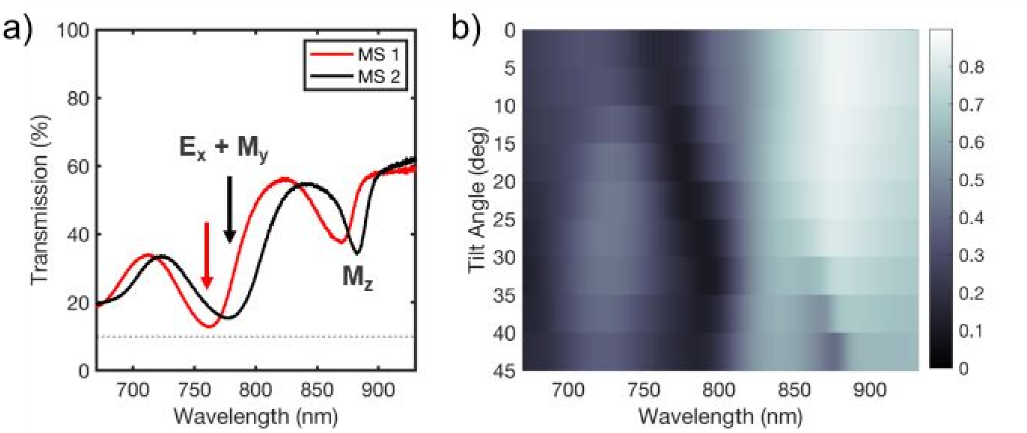

Figure S6. a) Experimentally measured transmission spectra for two metasurfaces at the angle of incidence of 45°: for each metasurface the electric dipole, E_x and magnetic dipole, M_y overlap and create a single dip in transmission shown by the red and black arrows. **b)** Metasurface (MS 2) transmission spectra for different angles of incidence. Minimum transmission is reached at an incidence angle of ~30°.

4. Measurements of THz Pulse Generation

4.1 Experimental Setup

Generation of THz pulses from the metasurface and thin GaAs layers is measured using a THz emission spectroscopy setup. The sample is excited by 100 fs near-infrared (NIR) pulses from a Ti:Sapphire laser as shown in Figure S7. The NIR laser beam (780nm, repetition rate 80 MHz) is split by a 1/100 beam splitter into two beam paths - the pump and probe beams. The pump beam (500 mW) is directed through a half-waveplate and focused onto an ~100 μm spot on the metasurface tilted at 45° to the beam axis. The waveplate is used to adjust the polarization angle of the pump beam, θ. A 650 μm thick wafer of (100) GaAs is placed after the metasurface to block the pump beam while allowing the THz pulse to pass through it.

The intensity of the probe beam is adjusted to 100 μW and the beam is focused on a photoconductive antenna (PCA) detector [6], which is positioned ~8 mm away from the metasurface without any focusing THz optics to avoid any pulse distortion. To detect THz pulses, the pump beam is modulated at 1.73 kHz by a mechanical chopper, and the PCA detector current is pre-amplified and demodulated by a lock-in amplifier at the chopping frequency. The lock-in amplifier time constant is 300 ms. By changing the delay between the pump and probe beam, the THz field is sampled in time to build up the full THz pulse waveform from which spectral amplitude is obtained using Fourier transform.

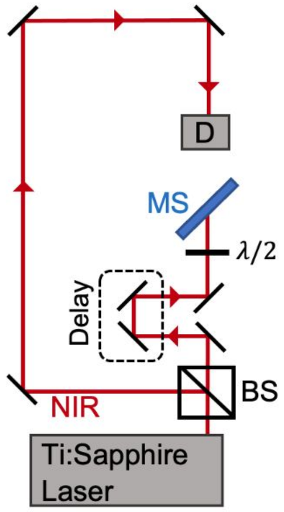

Figure S7. Schematic of THz emission spectroscopy setup.

To verify if birefringence in the sapphire substrate affects the dependence of THz generation on the incident polarization, the generated THz pulses from a metasurface were measured in two configurations: firstly, when the NIR pulse passes through the sapphire substate first, and secondly when the sample is flipped, so that the NIR pulse hits the metasurface first. The results of this can be seen in Figure S8. The waveform shape, THz field amplitude and polarization dependence are very similar between the two cases. The only difference is in the time of arrival of the THz pulse: it arrives later in the second case, as the THz pulse slows down while passing through the sapphire substrate, which has a higher optical index at THz frequencies in comparison to the NIR frequencies.

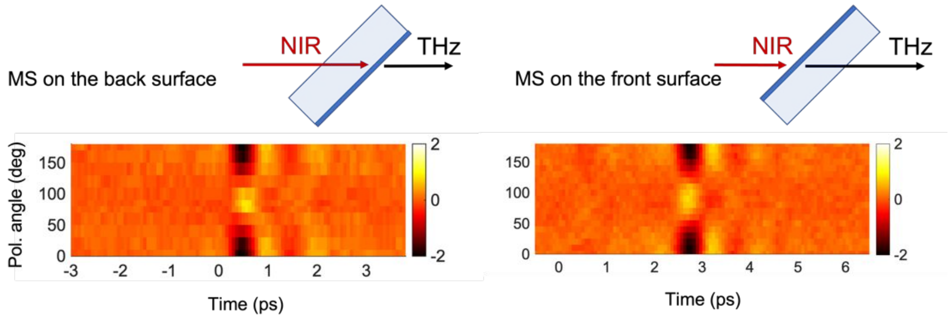

Figure S8. The polarization dependence of the emitted THz field measured from the same metasurface when the NIR light passes through the substrate first, vs when the NIR hits the metasurface first.

4.2 THz Generation Measurements of thin GaAs Layers

Figure S8 shows the full time-domain waveforms measured from the 55nm and 275nm GaAs layers for varying incident polarization ($\theta = 0°$ - $\theta = 180°$), and for two different orientations about the surface normal ($\alpha = 0°$ and $\alpha = 90°$). For both thicknesses, the THz waveforms have the same polarity and time domain shape. However, a different polarization dependence can be seen for the different sample orientations. For the 55nm case, the two sample orientations give similar dependence on polarization with a slightly higher THz peak-to-peak amplitude for the first sample orientation (Fig. S9b,c). However, for the 275 nm sample, the second sample orientation shows a much higher polarization dependence – with over 2x difference in field amplitude between p- and s-polarizations. This complicated dependence on layer thickness, incident field polarization and crystal orientation cannot be explained by photocurrent and volume nonlinearity mechanisms alone, suggesting the contribution of an alternative THz generation mechanism, which we attribute to surface nonlinearity.

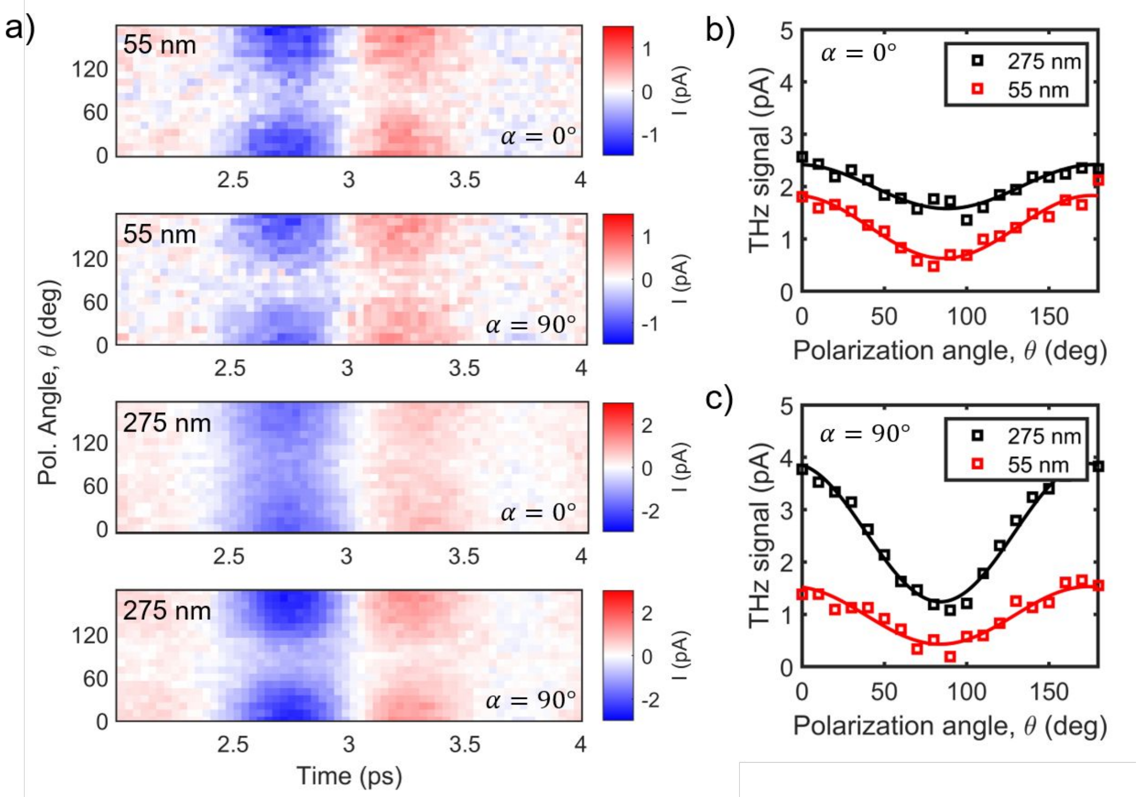

Figure S9. A) Time-domain waveforms measured with varying incident field polarization angle from GaAs layers with thicknesses 55 nm and 275 nm. Measured for two different orientations about the surface normal (see Fig. S1) b) THz peak-to-peak amplitude measured with varying incident field polarization angle from GaAs layers with thicknesses 55 nm and 275 nm. c) Same as part b, for the sample oriented at 90 degrees about the surface normal with respect to the orientation in part b.

5. Dependence of THz Generation on Metasurface Geometry

To evaluate how sensitive the THz generation process is to small variations in the metasurface geometry, the THz pulse amplitude is measured for multiple fabricated metasurfaces, each with different wide and narrow bar widths. Both parameters affect the wavelengths of metasurface modes.

Figure S10 shows peak-to-peak amplitude of emitted THz pulses for a set of metasurface bar widths. Maximum THz pulse amplitude is measured for metasurfaces with the narrow bar of 50-60 nm and the wide bar of 110-120 nm. The THz field amplitude drops to ~50% of the maximum value for other metasurfaces in the set. For comparison, THz pulses emitted from a uniform layer of GaAs of the same thickness (160 nm) are ~25% of the maximum amplitude generated from the metasurface.

The difference in THz emission amplitude from different metasurface geometries can be caused by a combination of factors. Most significantly, when the resonance wavelength of the metasurface is detuned from the excitation beam wavelength, we see a reduction in absorption at the excitation

wavelength, resulting in a reduction in number of photoexcited carriers and thereby a reduced THz field amplitude. For example, in numerical simulations, we see a reduction in absorption of 36% at the excitation wavelength when the narrow bar width is tuned from 50nm to 80nm, as shown experimentally in Figure S9. Although further analysis of these results is beyond the scope of this work, we point out that the observed effect of THz emission enhancement from GaAs metasurfaces is stable for 10-15% variation in the size of metasurface nano-scale features.

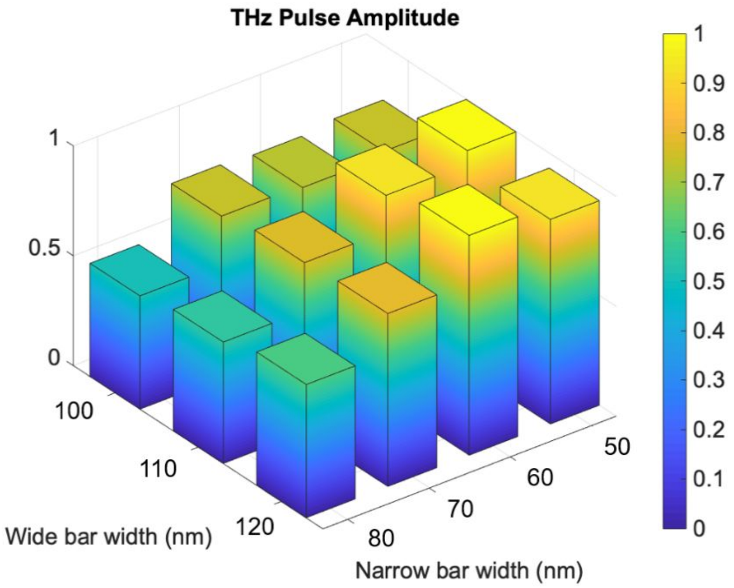

Figure S10. Normalized peak-to-peak THz pulse amplitude for 12 metasurfaces with varying widths of the narrow bar (50 – 80 nm) and wide bar (100 – 120 nm).

6. THz Generation due to Second-Order Nonlinearity in Metasurface Volume

6.1 Induced nonlinear polarization

To estimate the contribution of second-order GaAs nonlinearity to the effect of THz pulse generation in a 160 nm thick layer of GaAs and in the metasurface, we calculate induced nonlinear polarization using the second-order susceptibility tensor and the numerically simulated electric fields. First, full vectorial field distribution in the thin GaAs layer and the metasurface are found using the FDTD solver and the simulation setup as described in Section S1. The simulated field components in the metasurface reference frame, $E_{x'}$, $E_{y'}$ and $E_{z'}$ are then projected onto the crystal axes x_c = [100], y_c = [010] and z_c = [001] to obtain the field components in the crystal reference frame, E_{cx}, E_{cy} and E_{cz} for both *s*- and *p*-polarized illumination (the orientation of crystallographic axes is shown in Fig. S1). Then, the field

components in the crystal reference frame, E_{cx}, E_{cy}, and E_{cz} are obtained for an arbitrary polarization angle θ as follows:

$$E_{cx} = -E \sin\beta \sin(\pi/4) \cos\theta + E\sin(\pi/4)\sin\theta \quad (6.1)$$

$$E_{cy} = E\sin\beta \sin(\pi/4)\cos\theta + E\sin(\pi/4)\sin\theta \quad (6.2)$$

$$E_{cz} = E \cos\beta\cos\theta, \quad (6.3)$$

where E is the amplitude of incident NIR wave, θ is the polarization angle (θ = 0° corresponds to *p*-polarized excitation and θ = 90° corresponds to *s*-polarized excitation), and the $\sin(\pi/4)$ multiplication factors are added to account for the orientation of metasurface axes x', y' and z' along the GaAs crystallographic directions [110], [-110] and [001], respectively.

For the incidence angle $\beta = 45°$, the expressions above simplify to:

$$E_{cx} = -a^2E\cos\theta + aE\sin\theta \quad (6.4)$$

$$E_{cy} = a^2E\cos\theta + aE\sin\theta \quad (6.5)$$

$$E_{cz} = aE\cos\theta, \quad (6.6)$$

where $a = \sin\left(\frac{\pi}{4}\right) = 1/\sqrt{2}$.

Having obtained the electric field components in the crystal frame for every point within the metasurface we now use the second-order nonlinear polarization tensor $\chi^{(2)}$ to calculate the induced non-linear polarization vector distribution P at the zero frequency using the equation:

$$\begin{bmatrix} P_{cx}(\omega=0) \\ P_{cy}(\omega=0) \\ P_{cz}(\omega=0) \end{bmatrix} = 2\varepsilon_0\chi^{(2)} \begin{bmatrix} E_{cx}^2 \\ E_{cy}^2 \\ E_{cz}^2 \\ 2E_{cy}E_{cz} \\ 2E_{cz}E_{cx} \\ 2E_{cx}E_{cy} \end{bmatrix} \quad (6.7)$$

where the susceptibility tensor for bulk GaAs:

$$\chi^{(2)} = \begin{bmatrix} 0 & \cdots & 0 & d_{14} & 0 & 0 \\ \vdots & \ddots & \vdots & 0 & d_{25} & 0 \\ 0 & \cdots & 0 & 0 & 0 & d_{36} \end{bmatrix} \quad (6.8)$$

with all non-zero tensor elements equal to each other: $d_{14} = d_{25} = d_{36}$ [].

When the metasurface is excited, the amplitude of each field component rises rapidly first, following the temporal profile of the excitation pulse, and then decays following the resonance decay of the excited modes: $E_{ci} = E_{ci}(t)$. As a result, the induced non-linear polarization also varies in time:

$P_{ci} = P_{ci}(t)$. The evolution of induced polarization gives rise to emission of THz pulses, where the electric field amplitude is proportional to the second-order derivative of the induced polarization [7]:

$$E_{THz} \propto \frac{d^2 P_c}{dt^2}. \tag{6.9}$$

To estimate the amplitude of THz pulses emitted in the forward direction and having the vertical orientation of the electric field vector (*p*-polarized), we sum up projections of the induced polarization components P_{ci} onto the vertical axis (y-axis in Figure 1 of the Article):

$$E_{p,THz} \propto -a^2 \frac{d^2 P_{cx}}{dt^2} + a^2 \frac{d^2 P_{cy}}{dt^2} + a \frac{d^2 P_{cz}}{dt^2}. \tag{6.10}$$

The expression above carries implicit dependence on the polarization angle θ and therefore allows us to find the dependence of THz pulse amplitude, $E_{p,THz}$ on the polarization angle θ.

Assuming that the temporal evolution of the nonlinear polarization $\frac{d^2 P_c}{dt^2}$ is similar for all polarization components everywhere within the metasurface, we can further simplify the expression for the amplitude of the THz pulse:

$$E_{p,THz} = \sum C(-a^2 P_{cx} + a^2 P_{cy} + a P_{cz}), \tag{6.11}$$

where C is a constant, and the sum symbol represents summation of local non-linear polarization over the metasurface unit cell.

6.2 THz pulse amplitude dependence on excitation polarization angle

We calculate the THz pulse amplitude according to the expression above for every polarization angle θ and the corresponding dependence of the pulse amplitude on θ is presented in Fig. 4 (see Article) for two cases: the metasurface (Fig. 4a) and a 160 nm uniform layer of GaAs (Fig. 4b). To facilitate comparison between the two cases, we normalized both functions to the amplitude of the THz field generated in the uniform 160 nm layer for the *p*-polarized excitation.

Using the approach outlined above, we can evaluate the THz pulse amplitude dependence on the polarization angle also for the metasurface and the uniform layer rotated 90° about the surface normal ($\alpha = 90°$ in Fig. S1). For the rotated uniform layer, the non-linear polarization changes its sign and, as a result, the THz pulse simply changes its polarity. In the case of metasurface however, both the crystallographic orientation and the metasurface orientation are rotated around the surface normal and, as a result, the metasurface exhibits a different electric field distribution and consequently the functional dependence of the induced polarization within the unit cell changes slightly. In particular, for the *s*-

polarized excitation, THz emission from the metasurface changes the polarity as well as the amplitude (by a factor of ~2) upon the metasurface rotation by 90° about the surface normal.

6.3 Analysis of THz generation mechanisms in a 160nm GaAs layer

In Figure 3 of the Article main text, we showed the measured THz emission dependence on the polarization angle θ for the metasurface and for a uniform 160nm thick GaAs layer of the same thickness. Here, we provide analysis of this dependence for the 160nm thick GaAs layer.

The THz pulse amplitude is maximum for the *p*-polarized excitation and, as the polarization is rotated, the amplitude decreases. Almost no emission at all is observed when it is excited with *s*-polarized light (see Article, Fig. 3a, black circles). When the crystallographic axes of GaAs are rotated by 90° around the surface normal (Fig. 3b), we still observe maximum THz field amplitude for the *p*-polarization, except the THz polarity is now reversed. Again, almost no emission is observed for *s*-polarized light. Although the change in polarity upon crystal rotation is consistent with the second-order nonlinearity within the GaAs layer, the lack of generated THz polarization for *s*-polarized excitation is completely unexpected.

Using our calculations of nonlinear polarization, described in Section 6.1 and presented in Figure 4 of the main text, we predict that the THz field due to the second order non-linearity in the thin layer volume switches polarity for the *s*- and *p*-polarized excitations. In both cases, we predict the THz pulses to have similar absolute amplitudes. When the crystal is rotated, the THz field polarity is changed again, but the absolute amplitudes still remain similar for the *s*- and *p*-polarized excitations.

Therefore, the lack of significant THz pulse emission for the *s*-polarized excitation in the experiment cannot be explained by a second-order nonlinearity mechanism within the GaAs volume; nor can it be explained by adding the effect of drift photocurrents, as the drift photocurrents are independent of the crystal orientation, and the number of excited charge carriers vary only slightly between the *s*- and *p*-polarized excitations. We therefore conclude that the generation mechanism must have a different polarization and crystal orientation dependence to that of the second order nonlinearity in the crystal volume. It is important to note that nonlinear THz generation is still present in the layer volume, however an additional mechanism destructively interferes with it for *s*-polarized excitation causing the lack of THz emission observed.

6.4 Induced nonlinear polarization in metasurface unit cell

Equation 6.11 is used to calculate the spatial maps of induced nonlinear polarization shown in Figure 5 of the Article. Figure S11 summarizes graphically how the plots of the metasurface nonlinear polarization in Figure 5 are obtained from the calculated nonlinear polarization over the whole 3D metasurface area. The projection of nonlinear polarization onto the detection axis ($E_{p,THz}$) is summed over the *xy*-plane at each *z* position in the unit cell defined by the FDTD mesh size (10 nm for the thin layer, 5 nm for the metasurface).

In Figure S11a, the nonlinear polarization is shown as a function of vertical position *z* in the metasurface, and is calculated for varying incident polarization angle, θ. The non-linear polarization for each vertical (z) position is summed and then normalized across the x-y plane, and the dependence on

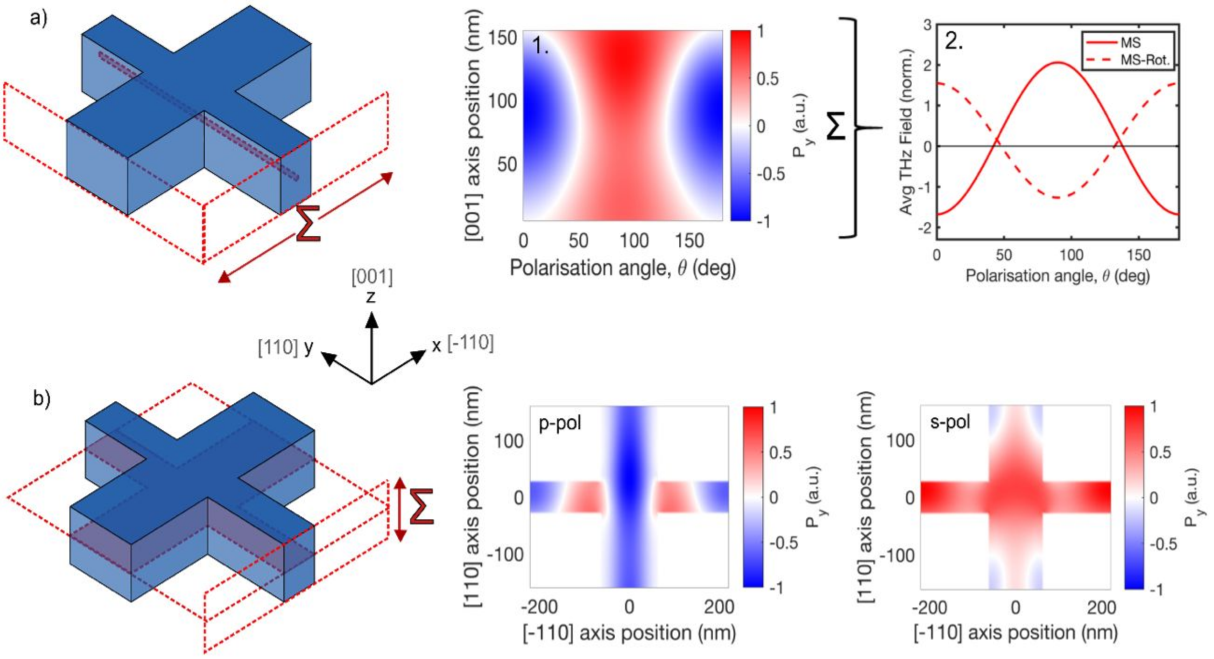

Figure S11: Non-linear polarization within metasurface unit cell: a) The nonlinear polarization as a function of vertical position is calculated for incident field polarizations 0° - 180° and summed across all x and y positions to obtain plot 1 (Fig.4b of main text). Plot 2 (Fig. 4b of main text) is obtained by summation over the vertical coordinate in plot 1 and normalizing it to the maximum nonlinear polarization of the thin GaAs layer (p-polarized). This is calculated for two rotations of the metasurface ($\alpha = 0°$ solid line, $\alpha = 0°$ dashed line). b) The nonlinear polarization in a horizontal (xy-plane) of the metasurface is calculated by summing over all z positions. This is calculated for p-polarized ($\theta = 0°$) incident field (Fig. 4c of main text) and s-polarized ($\theta = 90°$) incident field (Fig. 4d of main text). Axes show how the metasurface x,y,z axes correspond to the crystal axes [-110],[110],[001].

polarization angle is calculated using the same method as for the data in Figure 4 (shown in Fig. S11a-2).

In Figure S11b the nonlinear polarization is displayed as a function of x-y position in the metasurface for *p*- and *s*-polarized excitations (middle and right panel respectively). For each position in the x-y plane, the polarization is summed over the vertical axis (z-direction). The polarization function P_y (x,y) in the xy-maps is normalized to the largest absolute polarization value in the corresponding map.

References

1. Lumerical, "Lumerical FDTD," https://www.lumerical.com/products/fdtd/.
2. Lumerical, "Broadband Fixed Angle Source Technique (BFAST)," https://support.lumerical.com/hc/en-us/articles/360034902273-Broadband-Fixed-Angle-Source-Technique-BFAST-.
3. Liang, B.; Bai, M.; Ma, H.; Ou, N,; Miao, J. Wideband Analysis of Periodic Structures at Oblique Incidence by Material Independent FDTD Algorithm, IEEE Trans. Antennas & Propagation, **2014**, *62* ,1, 354 - 360.
4. Piper, J. R.; Liu, V.; Fan, S. Total Absorption by Degenerate Critical Coupling. *Appl. Phys. Lett.* **2014**, *104*, 251110.
5. Mitrofanov, O.; Hale, L. L.; Vabishchevich, P. P.; Luk, T. S.; Addamane, S. J.; Reno, J. L.; Brener, I. Perfectly Absorbing Dielectric Metasurfaces for Photodetection. *APL Photonics* **2020**, *5*, 101304.
6. Hale, L.L.; Harris, C.T.; Luk, T.S.; Addamane, S. J.; Reno, J. L.; Brener, I.; Mitrofanov, O. Highly efficient terahertz photoconductive metasurface detectors operating at microwatt-level gate powers, Opt. Lett. **2021**, *46*, 3159-3162.
7. Chuang, S. L.; Schmitt-Rink, S.; Greene, B. I.; Saeta, P. N.; Levi, A. F. J. Optical Rectification at Semiconductor Surfaces, Phys. Rev. Lett. **1992**, *68*, 102.
